# Social determinants of health: the need for data science methods and capacity

**DOI:** 10.1016/S2589-7500(24)00022-0

**Published:** 2024-04

**Authors:** Rumi Chunara, Jessica Gjonaj, Eileen Immaculate, Iris Wanga, James Alaro, Lori A J Scott-Sheldon, Judith Mangeni, Ann Mwangi, Rajesh Vedanthan, Joseph Hogan

**Affiliations:** School of Global Public Health (RC, JG), Tandon School of Engineering (RC), and Grossman School of Medicine (JG, RV) New York University, New York, NY 10003, USA; College of Health Sciences, Moi University, Eldoret, Kenya (EI, IW, JM, AM); National Cancer Institute (JA), and National Institute of Mental Health (LAJS-S), National Institute of Health, Bethesda, MD, USA; Brown University School of Public Health, Brown University, Providence, RI, USA (JH)

Social determinants of health (SDoH) include forces, systems, and conditions that shape the environments in which people are born, grow, work, live, and age. Racism and climate change, for example, affect quality of life and other health outcomes through structural factors, including economic policies, social norms, and other factors that shape environments and consequently the behaviours of people. Indeed, mitigating health inequities requires attention to their root causes and adjacent factors. SDoH can have a greater effect on quality of life and other health outcomes than health-care spendings or lifestyle choices alone.^1,2^ Accordingly, we need to inform analytical models (eg, those used to assess the effect of exposures or interventions on health outcomes) to account for, analyse, and implement interventions on SDoH, such as greenspace improvements.^3^ In this Comment, we highlight three challenges to measuring and analysing SDoH for which data science—a cross-disciplinary set of skills to make judgements and decisions with data by using it responsibly and effectively—can be harnessed. The three challenges listed are briefly introduced and elaborated on, including clear examples of data science approaches to address: data necessary for capturing the exposure of interest at multiple levels appropriately are not always available nor easy to measure; SDoH are distal to individual health outcomes compared to biomedical determinants such as comorbidities; and the distal placement of SDoH in relation to health outcomes results in requires long periods of time to observe their effect (in some cases over decades or generations).

The complex interplay of SDoH at individual, community, societal, and policy levels, and how these determinants operate to affect health outcomes is shown in the figure. The first challenge is that data necessary for capturing the exposure of interest at multiple levels (eg, social, policy, etc) appropriately are not always available or easy to measure. For example, frameworks of health have been enhanced to include forms of discrimination (eg, racism),^4^ but these phenomena are difficult to quantify. Previous work makes use of observable but imperfect proxies of discrimination, such as social position (eg, refering to an individual’s or a group’s place or status by using race, instead of the acting force of racism) or variables (eg, population segregation) that might not be at scales relevant to the lived experience of all individuals.^5^ Data science methods can be used to create measures of discrimination from unstructured data without a specified format (eg, clinic notes) and other concepts not easily captured or readily available in existing databases. For example, natural language processing and machine learning along with precise geolocation of social media data have been used to ascertain discriminatory climate—one aspect of structural discrimination, embodied by a particular place.^6^ Creating such measures assures improved understanding of the effects of multiple forms of discrimination on health by complementing existing data types, such as perceived discrimination from surveys, segregation from census data, or protections based on policy data. The increased granularity over time (eg, daily or sub-daily) of such data can also add an increased number of observations that can capture changes at a higher cadence than through data requiring more resources for collection, such as surveys. Other examples of ascertaining SDoH include use of natural language processing to extract social determinants (eg, housing insecurity) from unstructured data in electronic health records and deep learning to extract environmental factors from satellite data. The data generation process for such observational data should be carefully inspected and evaluated for any hidden biases or imbalances, such as selection or representation biases. This inspection can be done through exploratory data analysis and summary statistics with attention to demographic variables, or qualitative analyses of the included information. Furthermore, data inspection, cleaning, standardisation, and organisation practices, which are also key elements of data science methodology, are needed to link SDoH to other data. By incorporating SDoH into analytical models statistical assumptions (eg, around conditional mean or independence), which remove group-level variance incurred by neighbourhood and other SDoH from the estimation problem, can also be eased. Using unstructured data to represent SDoH can thus better capture and be inclusive of the heterogeneity of effects experienced by different people.

The second challenge is in terms of modelling; SDoH are distal to individual health outcomes compared with biomedical determinants, such as comorbidities. Consequently, there can be several intermediate factors interceding the relationship between SDoH and health outcomes. These added junctures introduce complexity (non-linear interactions), confounding, and feedback pathways. The relationship between income dynamics and health outcomes illustrates this issue—income influences health through complex pathways (eg, availability of resources and time to devote to healthy behaviours), while health can also affect income earning ability. Assuming a conceptual model describing the pathways between variables is properly specified, machine learning can be helpful for capturing complex relationships between observed variables and enabling flexibility between model components.^7^ Complex, multifactorial causal pathways do not lend themselves to testing with randomised experiments, further motivating the need for observational data analysis approaches to analyse large sets of variables and effects together. Data science approaches, such as agent-based models and other complex systems dynamic computational models that leverage statistical methods in concert with data-driven methods, have also proven useful given the large number of variables and interactions in SDoH analyses.^8^ These relationships can be difficult to capture with standard regression models, such as parametric regression, and thus might not adequately be ascertained using traditional statistical methods.

A third challenge is that the distal placement of SDoH in relation to health outcomes requires long periods of time to observe their effect (in some cases over decades or generations). For example, while in a given geographical area lower availability of fresh produce, combined with a high concentration of fast-food outlets, and few recreational opportunities leads to poorer nutrition and physical activity;^9^ subsequent chronic diseases can take decades to appear. Longitudinal datasets that include necessary variables from such long time periods are rare and, if available, concentrated in high-income and well-resourced countries. This absence of long-term data challenges our ability to model and predict processes at relevant time scales in a broader set of global places. The required data collection methods (ie, denominator-based representative surveys) are resource-intensive. Data that might not have been generated for health purposes (eg, databases providing digital traces from mobile phones, the Internet, insurance claims, or web-connected devices) and with appropriate privacy protection (including but not limited to de-identification) can offer longitudinal insight. Examples include sleep data from mobile phones, food consumption behaviours from in-store grocery store card transactions. These data have the added advantage of being high in granularity with respect to place and time (eg, sub-neighbourhood and sub-daily).^10^ Given appropriate model and population considerations, like assuring that the population represented is appropriate and data complies with individual privacy and security laws, data management, linkage, and feature generation methods can be applied.

The important potential contribution of data science methods has been identified in all three challenges; the acquisition, integration, and coherent analysis of a holistic set of determinants, is only made possible with the involvement of individuals who have substantive knowledge of SDoH and their action on health outcomes in the appropriate context. To accomplish this goal effectively, we need to enable individuals with contextual knowledge to identify, capture, and model SDoH with data science methods. This gap motivates the need for local data science expertise and cross-disciplinary skills combined with the experience, culture, and values of the local residents. Indeed, there is a need for expanding the cadre of data scientists to be inclusive of people of a variety of backgrounds and places. For example, the US National Institutes of Health Common Fund Program in Data Science for Health Discovery and Innovation in Africa Initiative includes statistical, computing, and social science training with a focus on SDoH (via the NYU-Moi Data Science for Social Determinants Training Program) to elevate data science efforts domestically and globally. The use of data science to integrate SDoH has cross-cutting relevance across communicable and non-communicable diseases, trauma and injury, and maternal and perinatal health research, practice, and policy. Place-based, demographical, and disciplinary diversity in data scientists who can work with communities, policy makers, and other stakeholders to address the three challenges described, will improve comprehensive accounting of factors in the causal chain connecting exposures to health outcomes, shifting the focus from individualising health problems to a more holistic approach. This approach will more accurately capture the heterogeneity of effect–outcome relationships experienced by a diverse population and ultimately make future analyses, interventions, practices, and policies inclusive of diverse populations.

## Figures and Tables

**Figure:**
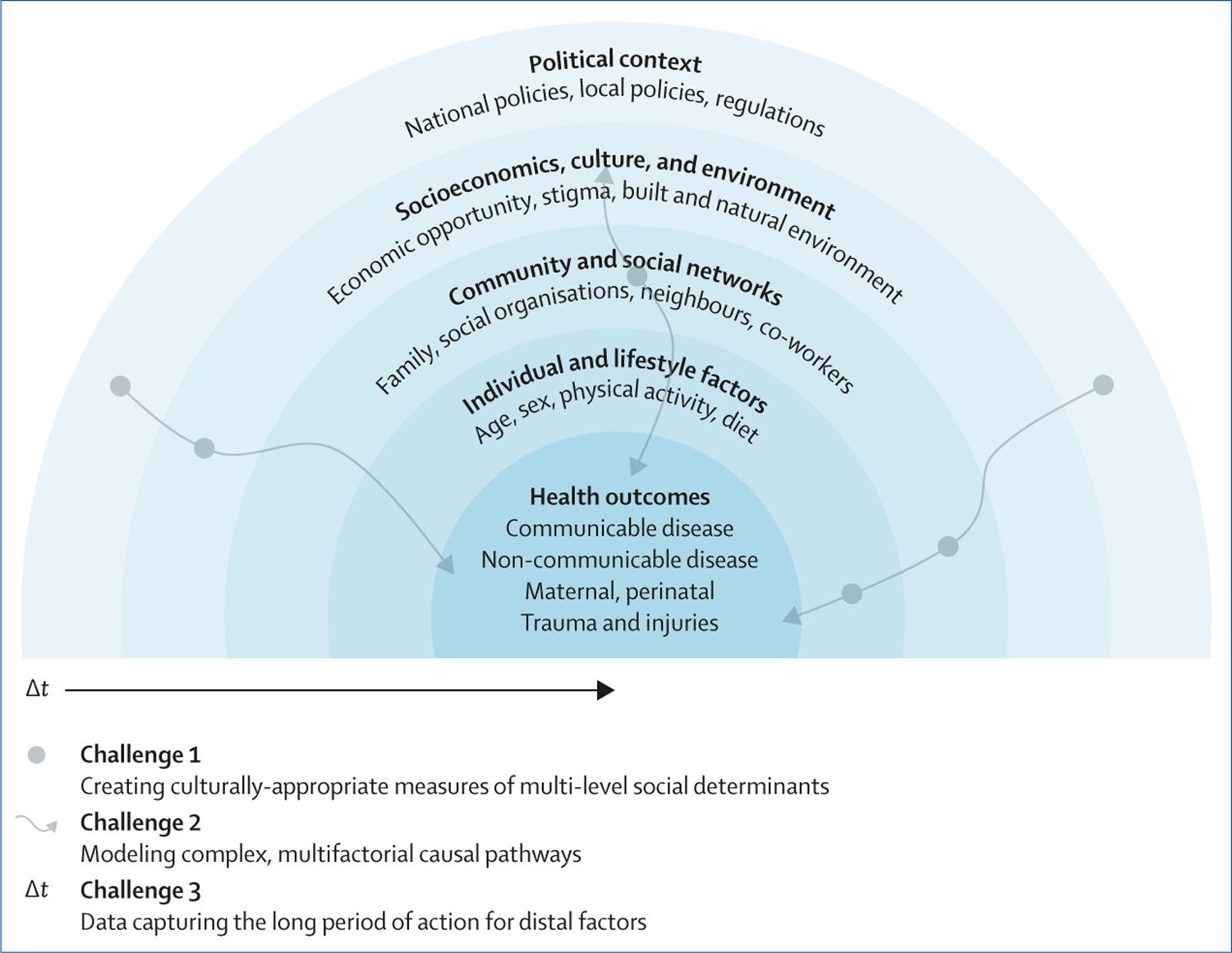
Illustration of the mode of action of social determinants of health on health outcomes and challenges of including SDoH in measurement and analysis The concentric blue circles depict social determinants influencing health outcomes, guiding the application of data science methods. Three challenges are highlighted: capturing the exposure of interest at multiple levels (eg, individual, neighbourhood, and national) in a culturally appropriate manner, capturing complex relationships between variables and enabling flexibility between model components, and considerations of the time required to observe the impact of an exposure on a health outcome endpoint. Individuals with substantive knowledge of Social Determinants of Health (SDoH) and their effect on health outcomes in the appropriate contexts need to be equipped with data science skills to address each of these challenges. The complex pathways and exposures at different levels are illustrated by grey wavy arrows and circles, respectively, reflecting the diversity involved in various analyses.
